# A 25 year nationwide analysis of mortality trends from cerebrovascular diseases in patients with lung cancer (1999–2023)

**DOI:** 10.1097/MS9.0000000000005165

**Published:** 2026-07-29

**Authors:** Sarim Hassan Shahab, Aown Abbas, Siddique Ahmed, Hadia Ghazala Masood, Faria Ali, Yazdan Sajid, Warda Imran, Ilsa Fatima, Amir Rafiq, Abubakar Nazir, Awais Nazir, Muhammad Zeeshan, Mobeen Z. Haider, Ahmed Jamal Chaudhary, Ghulam Mujtaba Ghumman, Naseer Khan

**Affiliations:** a Department of Internal Medicine, Nishtar Medical University, Multan, Pakistan; b Department of Medicine, Nishtar Medical University, Multan, Punjab; c Department of Medicine, Mohtarma Benazir Bhutto Shaheed Medical College, Mirpur, AJK, Pakistan; d Department of Medicine, Dow Medical College, Karachi, Pakistan; e Department of Internal Medicine, The Jewish Hospital – Mercy health, Cincinnati Ohio, USA; f Department of Internal Medicine, Oli Health Magazine organization, Kigali, Rwanda; g Department of Internal Medicine, King Edward Medical University, Lahore, Pakistan; h Department of Internal Medicine, Allama Iqbal Medical College, Lahore, Pakistan; i Department of Cardiology, West Virginia University, Morgantown, WV, USA; j Department of Medicine, DMC Sinai-Grace Hospital, Detroit, Michigan, USA; k Department of Internal Medicine, Mercy Health Saint Vincent Medical Center, Toledo, Ohio, USA; l Department of Cardiology, University Of Cincinnati, Ohio, USA

## Abstract

**Introduction:**

The two leading causes of death worldwide are lung cancer and cerebrovascular disease (CVD). Due to complications related to the disease and therapy, patients with lung cancer are much more likely to have a stroke, particularly within 2 years after diagnosis. Results are still poor despite medical advancements, underscoring the necessity of comprehensive prevention and care.

**Methods:**

ICD-10 codes for lung cancer (C34) and cerebrovascular illness (I60–I69) were used to evaluate mortality data from CDC WONDER (1999–2023). When both illnesses were listed on death certificates, deaths were counted. Sex, race/ethnicity, area, and urban/rural status were used to compute age adjusted mortality rates (AAMRs). Joinpoint regression was used to evaluate trends, and *P* < 0.05 was considered significant.

**Results:**

Overall AAMR for CVD and lung cancer decreased from 2.19 to 1.44 per 100 000 (APC: −2.9; *P* < 0.0001) between 1999 and 2015, subsequently increased to 1.71 in 2023 (APC: +2.8; *P* < 0.0001). From 3.08 in 1999 to 1.98 in 2023, men’s rates were continuously higher than women’s (1.59 to 1.49). The AAMR was highest among NH Black people (it peaked at 2.32 in 2023), followed by NH Whites (1.68) and Hispanics (0.69). Rural areas had higher AAMR than Urban areas. Alaska (2.57), Vermont (2.46), and Mississippi (2.46) had the highest state level burdens, while Utah (0.64) and New Mexico (0.86) had the lowest, while the South and Midwest region saw the sharpest recent rises.

**Conclusion:**

Lung cancer mortality from CVD increased until 2023 after decreasing until 2015. The U.S. South, rural areas, Black adults, and men had the biggest burden. To lower stroke mortality in patients with lung cancer, targeted interventions are required, with a focus on addressing racial and rural disparities in care, prevention, and access.

## Introduction

*Lung carcinoma* is the most common and leading cause of cancer-related mortality worldwide, with an estimated *2.48 million new cases* and *1.82 million deaths* reported in 2022, accounting for approximately 18% of all cancer deaths globally[1]. Despite substantial advances in screening, molecular diagnostics, and targeted therapies, the prognosis remains poor; in most regions, the 5-year survival rate rarely exceeds 20%[2]. The disease burden is driven largely by tobacco exposure, although never-smoker lung cancer often adenocarcinoma is rising, particularly in Asia, due to environmental exposures such as air pollution[3]. The public health impact is profound, as lung cancer not only imposes a high mortality risk but also significantly diminishes quality of life due to the aggressive nature of the disease and its treatment-related toxicities.

HIGHLIGHTS

- *Shifting mortality trend:* Age-adjusted mortality from combined lung cancer and cerebrovascular disease dropped steadily from 1999–2015 but has risen again since, reaching 1.71 per 100 000 in 2023 (APC: +2.8%).
- *Marked disparities:* Men, non-Hispanic Black adults, rural residents, and those in the South and Midwest bore the highest burden, with state-level peaks in Alaska, Vermont, and Mississippi.
- *Need for targeted action:* Findings underscore an urgent need for tailored stroke-prevention and care strategies for patients with lung cancer, especially addressing racial and geographic inequities.

*Cerebrovascular disease (CVD*), encompassing both ischemic and hemorrhagic stroke, remains one of the leading causes of death and disability globally, with approximately *11.9 million incident strokes* and *7.3 million stroke-related deaths* reported in 2021[4]. Although age-standardized mortality has declined in many high-income settings, the absolute number of cases continues to grow due to aging populations and rising prevalence of vascular risk factors including hypertension, diabetes, obesity, and atrial fibrillation[5]. Stroke survivors often face long-term neurological deficits, cognitive impairment, and reduced functional independence, creating a sustained burden on healthcare systems and caregivers[6]. Importantly, environmental exposures such as air pollution contribute significantly to the global stroke burden, with an increasing share of cases occurring in low and middle-income countries[7].

The intersection between *lung carcinoma and CVD* has garnered growing attention, with multiple epidemiologic studies demonstrating that lung cancer patients face a *significantly elevated risk of fatal stroke* compared with the general population^[^8,9^]^. This excess risk is most pronounced within the first 1–2 years following a cancer diagnosis[10] and is influenced by factors such as advanced disease stage, certain histologic subtypes, older age, and treatment modalities particularly thoracic radiotherapy which has been associated with a fourfold increase in ischemic stroke incidence[11].

While stroke mortality rates have improved for the general population, outcomes in lung cancer patients remain disproportionately poor, reflecting both biological mechanisms such as cancer-associated hypercoagulability and the cardiotoxic effects of treatment[12]. With population aging and improved cancer survival, the absolute number of lung cancer patients at risk for cerebrovascular events is expected to rise, underscoring the need for integrated prevention and management strategies targeting this high-risk group.

Lung carcinoma and CVDs remain among the leading causes of morbidity and mortality in the United States, despite decades of public health progress. While each disease independently impacts millions of Americans annually, their co-occurrence has received limited epidemiological attention. Utilizing CDC WONDER, this analysis examines national mortality trends and demographic disparities in lung carcinoma and CVD from 1999 to 2023, aiming to inform prevention strategies and integrated clinical care models.

## Methods

### Study setting and population

In this observational study, mortality information from death certificate data was sourced from the CDC WONDER (Centers for Disease Control and Prevention Wide-Ranging Online Data for Epidemiological Research) database and reviewed from 1999 to 2023 for Cerebrovascular Disorders along and Lung Cancer (CVD-LC)-related mortality in adults using codes from the International Statistical Classification of Diseases and Related Health Problems-10th Revision (ICD-10) as follow: I60-I69 and C34. The same ICD have been described previously to identify CVD and LC in administrative databases, respectively^[^13,14^]^. This dataset contains information on the causes of death recorded in death certificates from all the 50 states and the District of Columbia and has been utilized in multiple prior studies to analyze trends in CVD-LC mortality. The Multiple Cause-of-Death Public Use records from death certificates were analyzed to identify CVD-LC related deaths, defined as cases where CVD-LC were mentioned anywhere on the death certificate, either as a contributing factor or as the underlying cause of death. For the analysis, CVD-LC deaths were considered when CVD (I60-I69) was identified as the underlying cause and LC (C34) was listed as an additional contributing factor on the death certificate or *vice versa*. In this analysis, adults were classified as individuals aged 25 years or above at the time of death. A comparable age threshold has been adopted in prior studies to define adult populations[15]. This research was exempt from local institutional review board approval as it utilized a deidentified, government-issued public use dataset and adhered to the STROBE (Strengthening the Reporting of Observational Studies in Epidemiology) guidelines for reporting.

### Data abstraction

The abstracted data includes population size, year, location of death, demographic variables, urban-rural designation, region, and state. Demographic data covered sex, age, and race/ethnicity. The site of death was categorized as medical facility (outpatient, emergency department, inpatient, death on arrival, or status unknown), home, hospice, or nursing home/long-term care. Race/ethnicity categories were defined as non-Hispanic (NH) White, NH Black or African American (AA), Hispanic or Latino and Other (NH American Indian or Alaskan Native, and NH Asian or Pacific Islander). Mortality counts and population estimates were extracted directly from CDC WONDER using standardized query parameters. Suppressed or unreliable counts (in accordance with CDC reporting standards) were handled according to database guidance, and age-adjusted mortality rates (AAMRs) were calculated using the direct standardization method to the 2000 U.S. standard population. No individual-level identifiers were available. This analysis is based on information recorded in death certificates and has been utilized in earlier studies of the WONDER database[16]. The National Center for Health Statistics Urban–Rural Classification Scheme was applied to categorize the population as urban [large metropolitan area (population ≥1 million), medium/small metropolitan area population 50 000–999 999)] or rural (population <50 000) according to the 2013 U.S. Census classification. Geographic regions were defined as Northeast, Midwest, South, and West based on U.S. Census Bureau standards[17].

### Statistical analysis

National patterns in CVD-LC related mortality were evaluated by AAMRs per 100 000 population for the period 1999–2023, categorized by year, sex, race/ethnicity, state, and urban-rural status, with 95% confidence intervals (CIs). AAMRs were computed by standardizing CVD-LC related mortality counts to the year 2000 U.S. population. National yearly trends in CVD-LC related mortality were assessed using the Joinpoint Regression Program (Joinpoint V 5.4.0.0, National Cancer1 Institute) to estimate the annual percent change (APC) and average APC (AAPC) with 95% confidence interval[18]. This approach detects significant temporal shifts in AAMR by applying log-linear regression models at points where changes occur. APC values were interpreted as increasing or decreasing when the regression slope differed significantly from zero, based on two-tailed *t*-tests. A *P*-value below <0.05 was considered statistically significant.

## Results

A total of 96 050 deaths with location information were recorded. The distribution by place of death was as follows: medical facilities accounted for the largest proportion with 38 579 deaths (40.17%), which included inpatient facilities (38 242 deaths), outpatient/ER settings (2135 deaths), dead on arrival cases (156 deaths), and facilities with unknown status (64 deaths). The decedent’s home was the second most common location with 26 241 deaths (27.32%), followed by nursing homes and long-term care facilities with 18 063 deaths (18.81%). Hospice facilities accounted for 7250 deaths (7.55%), while other locations represented 3688 deaths (3.84%), and deaths with unknown location were 211 (0.22%). This distribution shows that medical facilities were the predominant place of death, followed by home settings and long-term care facilities.

## Annual trends

**Figure 1.**
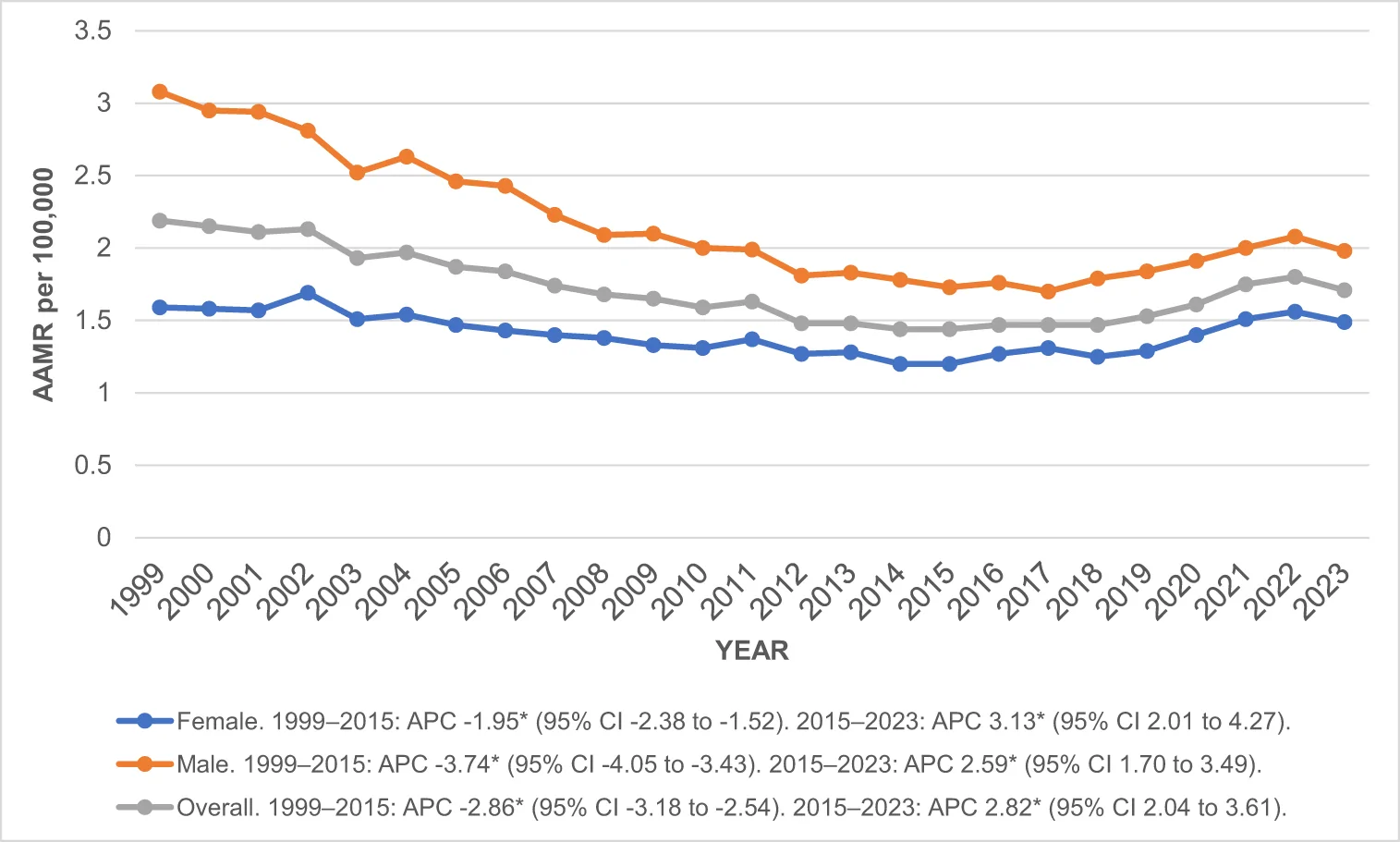
Gender disparities in mortality due to cerebrovascular disorders among patients with lung cancer.

Over the study period from 1999 to 2023, the AAMR showed significant changes with distinct trend periods:

The AAMR in 1999 was 2.19 per 100 000, which decreased to 1.44 per 100 000 in 2015. From 1999 to 2015, there was a consistent downward trend in the overall AAMR with an APC of −2.8610 (95% CI: −3.1762 to −2.5448; *P*-value: <0.000001). From 2015 to 2023, the trend reversed direction with the AAMR increasing from 1.44 to 1.71 per 100 000, showing an APC of 2.8192 (95% CI: 2.0376 to 3.6068; *P*-value: <0.000001). Over the entire study period from 1999 to 2023, there was an AAPC of −1.0034 (95% CI: −1.3137 to −0.6921, *P*-value: <0.000001). This reflects the overall downward trajectory despite the recent increase, indicating that the mortality rates in 2023, while higher than the lowest point in 2015, remained lower than the initial rates observed in 1999 (Fig. 1; Supplemental Digital Content Table 1, available at: http://links.lww.com/MS9/B214).

## AAMR stratified by sex

AAMRs were consistently higher among men compared to women throughout the study period. Among men, the AAMR decreased from 3.08 in 1999 to 1.73 in 2015 (APC: −3.7389, 95% CI: −4.0450 to −3.4318, *P*-value: <0.000001). From 2015 to 2023, the AAMR increased significantly to 1.98 (APC: 2.5888, 95% CI: 1.6950 to 3.4906, *P*-value: 0.000006). Among women, the AAMR decreased from 1.59 in 1999 to 1.20 in 2015 (APC: −1.9521, 95% CI: −2.3778 to −1.5246, *P*-value: <0.000001). From 2015 to 2023, the AAMR increased to 1.49 (APC: 3.1349, 95% CI: 2.0110 to 4.2712, *P*-value: 0.000010). Over the entire study period from 1999 to 2023, men demonstrated an AAPC of −1.6742 (95% CI: −2.0070 to −1.3403, *P*-value: <0.000001), while women showed an AAPC of −0.2850 (95% CI: −0.7210 to 0.1530, *P*-value: 0.201848) (Fig. 1; Supplemental Digital Content Table 1, available at: http://links.lww.com/MS9/B214).

## AAMR stratified by race/ethnicity

When stratified by race/ethnicity, AAMRs showed distinct patterns across racial and ethnic groups over the study period from 1999 to 2023.

The highest AAMR was observed in NH Black or AA adults. The AAMR decreased from 3.02 in 1999 to 1.78 in 2016 (APC: −3.1485, 95% CI: −3.6029 to −2.6919, *P*-value: <0.000001), followed by an increase to 2.32 in 2023 (APC: 4.0477, 95% CI: 2.3663 to 5.7569, *P*-value: 0.000057). Over the entire study period, NH Black or AA adults showed an AAPC of −1.1026 (95% CI: −1.6404 to −0.5618, *P*-value: 0.000067).

For NH White adults, the AAMR declined from 2.15 in 1999 to 1.40 in 2015 (APC: −2.7825, 95% CI: −3.1278 to −2.4359, *P*-value: <0.000001), followed by an increase to 1.68 in 2023 (APC: 3.0382, 95% CI: 2.0141 to 4.0726, *P*-value: 0.000004). Over the entire study period, NH White adults demonstrated an AAPC of −0.6797 (95% CI: −1.2598 to −0.4983, *P*-value: 0.000007).

In the NH Other race category adults, which includes American Indian or Alaska Natives, Asian and Pacific Islander or Native Hawaiian, the AAMR decreased from 1.23 in 1999 to 0.87 in 2015 (APC: −2.2862, 95% CI: −3.2040 to −1.3598, *P*-value: 0.000053), followed by an increase to 1.12 in 2023 (APC: 2.8421, 95% CI: 0.9103 to 4.8109, *P*-value: 0.005869). Over the entire study period, NH Other race category adults showed an AAPC of −0.6058 (95% CI: −1.4352 to 0.2305, *P*-value: 0.153203).

**Figure 2.**
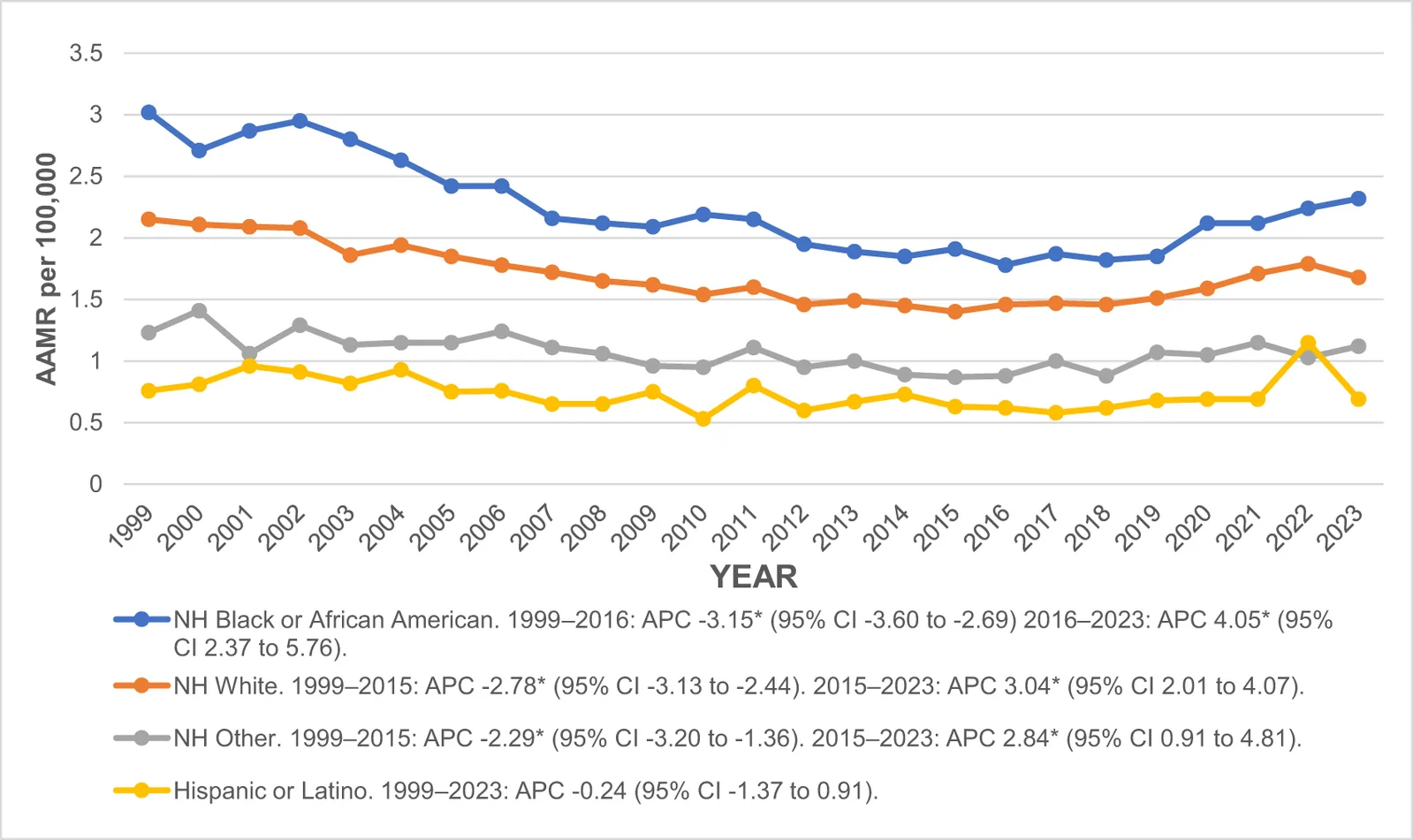
Racial disparities in mortality involving cerebrovascular disorders in patients with lung cancer.

The lowest AAMR was observed among Hispanic or Latino adults. The AAMR decreased from 0.76 in 1999 to 0.58 in 2017 (APC: −2.1521, 95% CI: −3.8869 to −0.3860, *P*-value: 0.019621), followed by an increase to 0.69 in 2023 (APC: 6.8308, 95% CI: −0.0440 to 14.1783, *P*-value: 0.051392). Over the entire study period, Hispanic or Latino adults demonstrated an AAPC of 0.0202 (95% CI: −1.9678 to 2.0485, *P*-value: 0.984261) (Fig. 2; Supplemental Digital Content Table 2, available at: http://links.lww.com/MS9/B214).

## AAMR stratified by geographical regions

**Figure 3.**
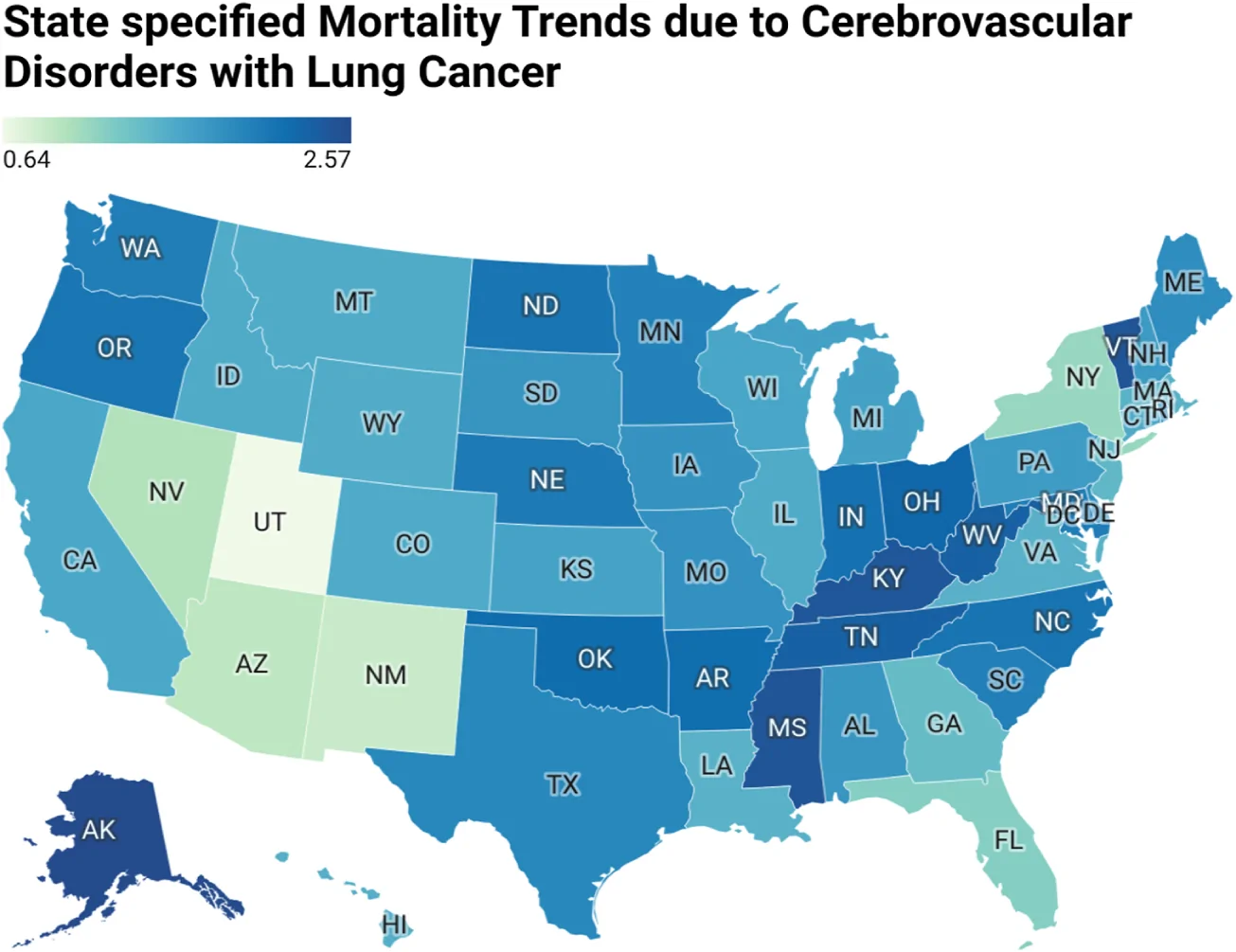
State-stratified mortality trends involving cerebrovascular disease among patients with lung cancer (1999–2020).

The highest AAMR was found in Alaska, with an AAMR of 2.57, followed by Vermont (2.46), Mississippi (2.46), Kentucky (2.44), and Tennessee (2.34). In contrast, states with the lowest AAMRs included Utah (0.64), New Mexico (0.86), Arizona (0.92), Nevada (0.98), and New York (1.12). This shows that the highest-burden states had AAMRs approximately 2-4 times higher than the lowest-burden states (Fig. 3; Supplemental Digital Content Table 3, available at: http://links.lww.com/MS9/B214).

In Urban Areas (Metropolitan Regions), the AAMR for mortality decreased from 2.14 in 1999 to 1.37 in 2014 (APC: −3.0876, 95% CI: −3.3948 to −2.7794, *P*-value: <0.000001), followed by an increase to 1.53 in 2020 (APC: 1.4487, 95% CI: 0.0982 to 2.8164, *P*-value: 0.036799). Over the entire study period from 1999 to 2020, urban areas demonstrated an AAPC of −1.8126 (95% CI: −2.2175 to −1.4061, *P*-value: <0.000001). This reflects a consistent downward trajectory with a recent increase.

**Figure 4.**
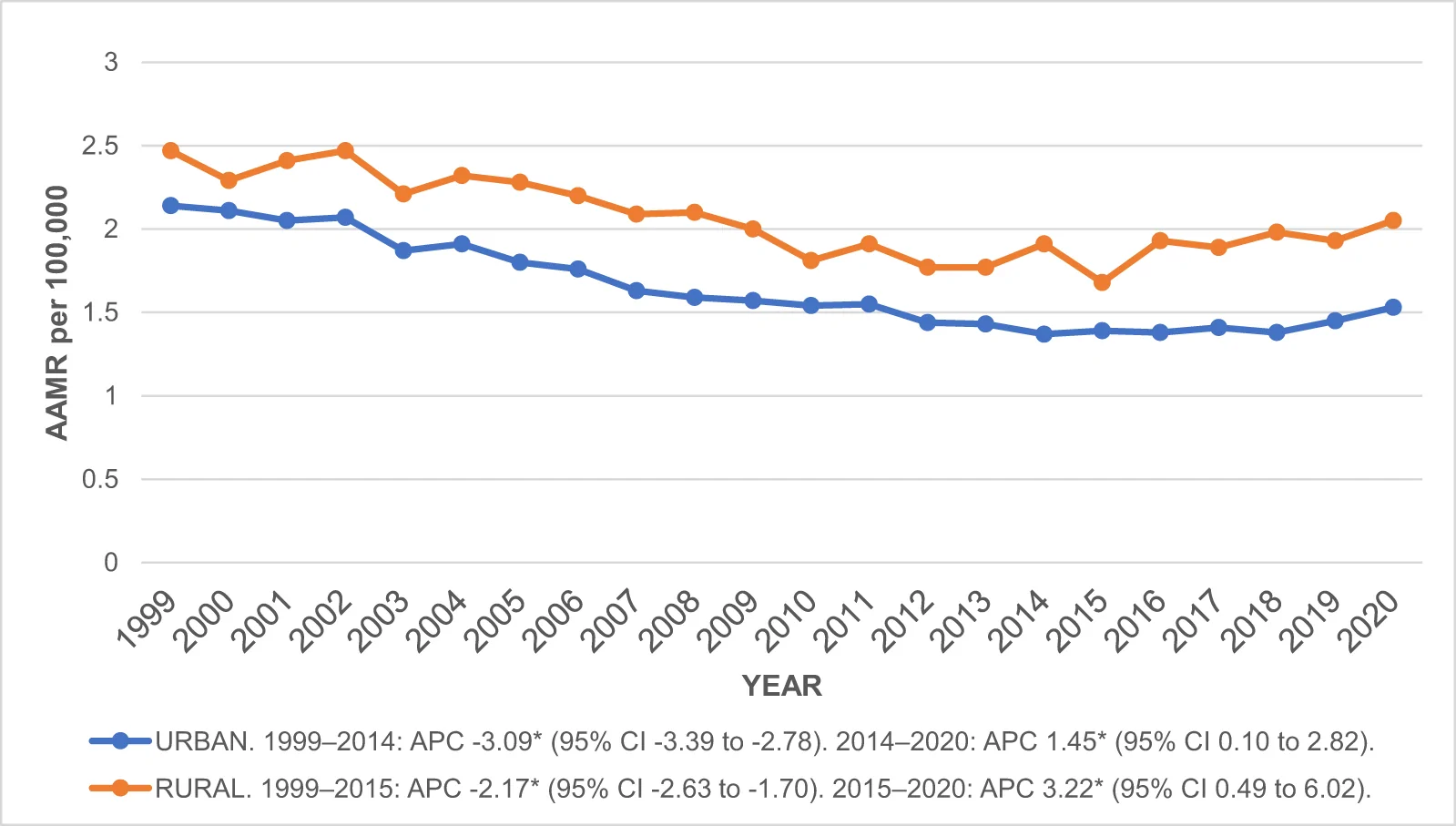
Urban/rural-stratified mort*a*lity trends involving cerebrovascular diseases in patients with lung cancer.

In Rural Areas (Nonmetropolitan Regions), there was a different pattern compared to urban areas. The AAMR decreased from 2.47 in 1999 to 1.68 in 2015 (APC: −2.1678, 95% CI: −2.6306 to −1.7027, *P*-value: <0.000001), followed by an increase to 2.05 in 2020 (APC: 3.2181, 95% CI: 0.4892 to 6.0210, *P*-value: 0.023227). Over the entire study period from 1999 to 2020, rural areas showed an AAPC of −0.9115 (95% CI: −1.5841 to −0.2343, *P*-value: 0.008413) (Fig. 4; Supplemental Digital Content Table 4, available at: http://links.lww.com/MS9/B214).

## Census region-level variation in AAMR

A clear difference in mortality was observed among U.S. Census regions in relation to deaths caused by the condition under study. The analysis used AAMR per 100 000 people and included 95% CIs to reflect the precision of these estimates.

**Census Region 3: South**. This region demonstrated the highest mortality progression with complex trends across multiple phases. The AAMR increased from 2.25 in 1999 to 1.54 in 2015 (APC: −2.6918; 95% CI: −3.1688 to −2.2125), followed by an increase to 1.91 in 2023 (APC: 3.6492; 95% CI: 1.4208 to 4.8924). Over the entire study period (1999–2023), the Southern region demonstrated an AAPC of −0.6225 (95% CI: −1.1022 to −0.1404; *P*-value: 0.011439).

**Census Region 2: Midwest**. This region showed significant mortality trends with notable changes over the study period. The AAMR declined from 2.37 in 1999 to 1.52 in 2016 (APC: −2.6089; 95% CI: −2.9650 to −2.2516), followed by an increase to 1.93 in 2023 (APC: 3.7829; 95% CI: 2.3918 to 5.1929). Over the entire study period (1999–2023), the Midwest region demonstrated an AAPC of −0.7864 (95% CI: −1.2349 to −0.3460; *P*-value: 0.000476).

**Census Region 1: Northeast.** The Northeast showed moderate mortality patterns with two distinct phases. The AAMR decreased from 2.00 in 1999 to 1.15 in 2016 (APC: −2.7254; 95% CI: −3.1797 to −2.2669), followed by an increase to 1.40 in 2023 (APC: 2.5883; 95% CI: 0.8548 to 4.3515). Over the entire study period (1999-2023), the Northeast region showed an AAPC of −1.2047 (95% CI: −1.7578 to −0.6485; *P*-value: 0.000023).

**Figure 5.**
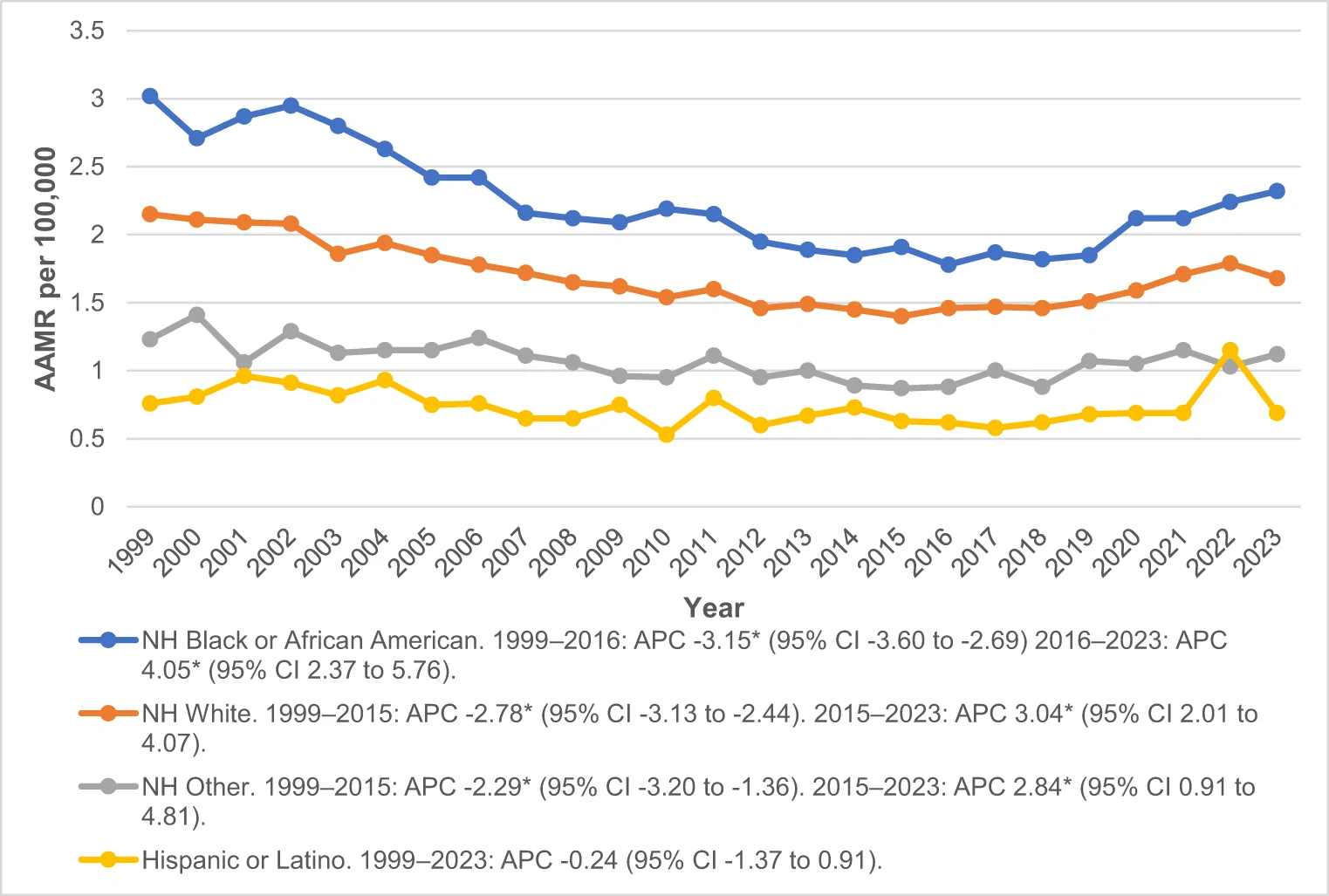
Census region-stratified mortality involving cerebrovascular diseases among adults with lung cancer.

**Census Region 4: West**. This region had the most pronounced mortality decline over the study period. The AAMR decreased from 2.02 in 1999 to 1.33 in 2014 (APC: −3.3915; 95% CI: −3.8216 to −2.9595), followed by an increase to 1.45 in 2023 (APC: 1.6913; 95% CI: 0.7644 to 2.6267). Over the entire study period (1999-2023), the Western region showed an AAPC of −1.5159 (95% CI: −1.9244 to −1.1058; *P*-value: <0.000001) (Fig. 5; Supplemental Digital Content Table 5, available at: http://links.lww.com/MS9/B214).

## Central illustration

**Figure 6.**
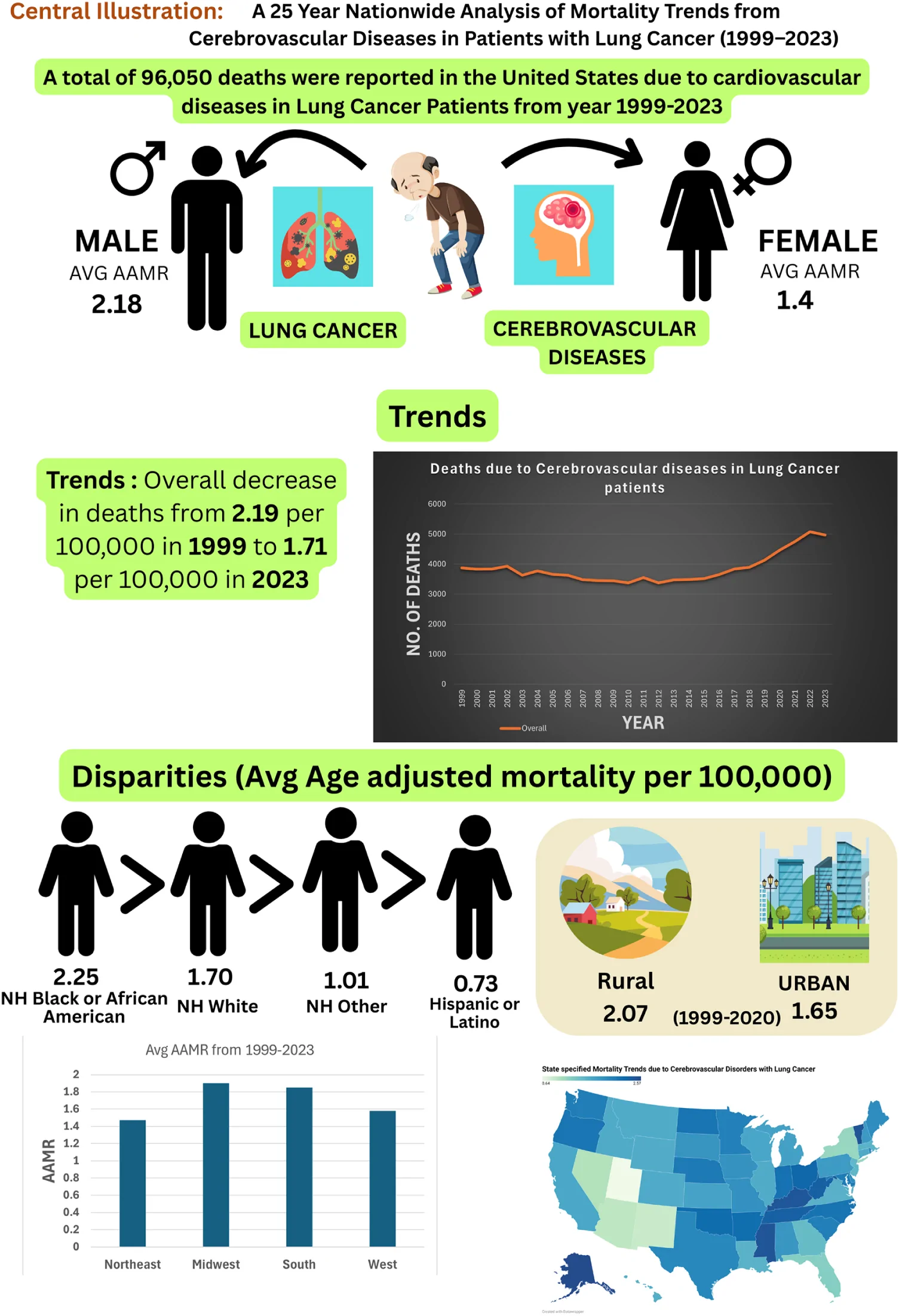
Central illustration.

A summarized Graphical Abstract is indicated in Figure 6 for quick review.

## Discussion

In this study of 1999 to 2023 mortality rate analysis of the data obtained from the CDC, we report several key findings. Age adjusted mortality rate (AAMR) decreased till 2015 and then showed an increase till 2023. But the overall trend remains downward. First, males had significantly higher mortality rate as compared to women. Second, Mortality was highest in NH Black or AA adults. Third, Rural Regions were disproportionally more affected as compared to Urban Areas. In the AAMR stratified by census region, the South showed the highest mortality progression with complex trends at multiple phases. State wise, the highest AAMR was found in Alaska followed by Vermont, Mississippi, Kentucky and Tennessee respectively. Targeted healthcare strategies and policy-level initiatives are necessary in view of the reported disparities.

CVDs and cancer are major health problems[19]. Lung cancer is the second most cause of cancer mortality[20]. Smoking is considered the major combined risk factor for lung cancer and for CVDs especially stroke^[^21,22^]^. Few previous studies have shown that patients with cancer of pancreas, lung, head and neck, oral cavity, and breast have high risk of cerebrovascular disorders Studies have also shown that brain metastasis occur in about 30% of patients with metastatic non-small cell lung cancer. There is high rate of intracranial hemorrhage in patients with metastasis than in patients without metastasis[23]. Implication of therapies such as cisplatin and radiation therapies during cancer increase the risk of cerebrovascular events. Additionally, cisplatin is associated with endothelial damage and thromboembolic events[24].

All these factors show the association of cerebrovascular disorders with lung cancer. However, there is still a gap in research regarding their association. Very few studies are there discussing the cause and reasons of cerebrovascular events in lung cancer. Future research should focus on studying their relation and role in in disease progression, underlying mechanisms, and potential strategies for prevention and management.

The reason for the overall decreased mortality trend in patients with cerebrovascular disorders and lung cancer may lie in improved screening techniques, which help in early diagnoses and treatment[25]. Public health initiatives such as smoking cessation programs and enhanced education regarding cerebrovascular disorders and the risk of lung cancer may also contribute to lowering the mortality rate[26].

Men had significantly higher mortality rates as compared to women. It may be because of smoking. Historically the rate of smoking was higher in men than in women. This may result in a higher risk of lung cancer in men than in women. But the overall decreasing trend is because smoking has been reduced in many regions. Moreover, men have a higher chance of developing lung cancer than women, and hormonal differences also play a role[27].

The complex interplay of socioeconomic statuses, health access, lifestyle, and cultural factors such as health beliefs and attitudes may be a cause of the highest mortality rate among NH Black or AA adults. Additionally, NH Blacks have a high mortality rate due to comorbidities such as hypertension and diabetes, which possibly can complicate the treatment of lung cancer and cerebrovascular disorders[28]. In the United States, there is still a clear gap in life expectancy between NH Black and NH White adults. On average, NH Black adults live about 4 years less than NH White adults. For example, in 2018, the life expectancy for Black adults was 74.9 years. By age 75, survival rates in 2020 were also lower for NH Black adults (10.8) compared with NH White adults (12.0)^[^29–31^]^.

According to our research, between 1999 and 2020, the number of lung cancer patients dying from cerebrovascular disorders decreased in both urban and rural locations. Nonetheless, rates were continuously higher in rural areas than in metropolitan areas. While rural areas showed more fluctuations and only partial improvement, urban areas showed a steadier decline. These variations point to persistent inequalities that are probably caused by the lack of resources and access to healthcare in rural areas, indicating the need for focused initiatives.

Among geographical regions, highest mortality rates were found among Alaska, Vermont, Mississippi, Kentucky, and Tennessee states, which can be attributed to poverty and lack of education. Such as Mississippi and Kentucky have highest poverty rate in the USA^[^32,33^]^. Rural areas such as Kentucky and Tennessee have fewer healthcare facilities and providers that can result in higher mortality rate^[^34,35^]^. Moreover, Alaska geographic isolation most possibly can hinder access to specialized medical care facilities[36]. South Region had the highest AAMR. In comparison to other regions, the South had the highest AAMR, most likely due to underdevelopment or because of a higher incidence of risk factors like smoking, obesity, hypertension, and restricted availability of specialist healthcare services.

The 25-year study period encompasses major changes in diagnostic practices, treatment strategies, and population demographics that may have influenced mortality trends. Advances in neuroimaging and lung cancer screening improved detection and risk stratification, while developments in targeted therapy, immunotherapy, radiotherapy, and modern stroke management likely contributed to earlier mortality reductions. Conversely, population aging, rising cardiometabolic risk factors, and improved cancer survival may have increased the number of individuals at risk for cerebrovascular events. Thus, the observed trends likely reflect both true epidemiologic shifts and evolving clinical practices.

The findings of our research could help us in developing interventions that address disparities in health outcomes across the different populations and communities. The higher mortality rate, especially due to poverty, can be managed by making transformations and addressing the underlying social determinants.

Recent advances in artificial intelligence, particularly large language models such as ChatGPT, are influencing biomedical research and data interpretation. A global bibliometric analysis highlights key concerns regarding data quality, interpretability, and ethical use in medical applications. Although our study relies on established epidemiologic methods, such tools may complement future large-scale population health research through enhanced pattern recognition and hypothesis generation[37].

These results should grab our attention to the areas where mortality rate is still high, and targeted public health strategies, resource allocation, and policies that can reduce these inequalities are needed. Patients with lung cancers should also be monitored carefully for cerebrovascular events. Our results also highlight the need for ongoing monitoring of lung cancer and cerebrovascular disorders. Future research should also be done to develop effective interventions that lower the mortality rate related to cerebrovascular disorders and lung cancer.

## Limitations

This study has several limitations. First, there is a chance that cerebrovascular disorders may have been misclassified or underreported as the cause of death for patients with lung cancer because the research was based on ICD coding and death certificate information. Second, a more complete picture of comorbidities and disease mechanisms could have been obtained from the dataset if it had included extensive clinical data, such as imaging investigations, laboratory results, treatment histories, or genetic information. Third, it was challenging to evaluate how treatment strategies might have affected mortality outcomes since there was a lack of information about cancer treatments and stroke-specific therapy. Moreover, although demographic data was recorded, important social determinants of health like socioeconomic position, education, and access to healthcare were left out, which could help to explain some of the discrepancies seen.

As an ecological analysis based on aggregated national data, this study is subject to ecological fallacy and cannot establish individual-level causal relationships. Variations in healthcare access, reporting practices, and regional certification standards may have introduced systematic bias that could partially explain geographic and racial disparities observed in our findings. These factors should be considered when interpreting temporal and subgroup comparisons.

## Conclusion

After an initial period of decline till 2015, the AAMR increased steadily till 2023. The highest AAMR was observed in men and among Black or AAs. Rural areas and South Region of the United-States were most affected. Reducing cerebrovascular mortality in patients with lung cancer should be one of the main goals of future public health initiatives, with a focus on rural communities where persisting inequities in access, management, and prevention continue to raise mortality rates.

## Data Availability

Not applicable.

## Transparency Statement

This document was prepared with a commitment to transparency, accuracy, and integrity in research. All data, methodologies, and analyses included in this research have been extracted from the CDC WONDER data base, an official website of the United States government. All data have been meticulously extracted and reviewed. Any potential conflicts of interest have been disclosed, and all efforts have been made to ensure that the findings presented are unbiased and based on robust evidence.

The authors have adhered to the ethical guidelines for research and publication, including maintaining objectivity and providing a clear and honest account of the research conducted. All sources of funding, if applicable, have been disclosed, and there has been no undue influence from sponsors or external parties in the preparation of this document. We encourage peer review and welcome constructive feedback to further enhance and refine the reliability of this work.
